# Impact of Adjuvant Nonavalent HPV Vaccination on Viral Clearance in HPV-Positive Women With and Without Excisional Treatment: A Retrospective Cohort Study

**DOI:** 10.3390/vaccines14020141

**Published:** 2026-01-29

**Authors:** Ali Deniz Erkmen, Kevser Arkan

**Affiliations:** Division of Gynecologic Oncology, Gazi Yaşargil Training and Research Hospital, Diyarbakır 21070, Turkey

**Keywords:** HPV vaccination, Gardasil 9, HPV clearance, LEEP, cervical intraepithelial neoplasia, histological regression, adjuvant vaccination

## Abstract

Background: Persistent infection with high-risk human papillomavirus (HPV) is the key driver of cervical carcinogenesis and post-treatment recurrence. Although excisional treatment effectively removes dysplastic tissue, it does not directly target viral persistence. While HPV vaccination is well established in primary prevention, its potential role as an adjuvant strategy in HPV-positive women, particularly with respect to viral clearance, remains incompletely defined. Methods: This retrospective cohort study included HPV-positive women with at least 12 months of follow-up who were managed at a tertiary gynecology clinic. Patients were stratified according to HPV vaccination status with the nonavalent vaccine (Gardasil 9) and excisional treatment status with loop electrosurgical excision procedure (LEEP). HPV clearance at 12 months was defined as the primary outcome, while histological outcomes were evaluated as secondary and independent endpoints. Analyses were performed in the overall cohort and stratified by LEEP status. Multivariable logistic regression was used to identify factors independently associated with HPV persistence, adjusting for baseline disease severity and clinical covariates. Results: A total of 935 HPV-positive women were included in the final analysis. Completion of the three-dose HPV vaccination schedule was associated with significantly higher HPV clearance rates at 12 months compared with no vaccination. This association was consistently observed in women who underwent LEEP as well as in those managed without excisional treatment. In multivariable analysis, HPV vaccination emerged as an independent protective factor against HPV persistence, whereas LEEP status itself was not independently associated with viral clearance after adjustment for baseline histological severity. Histological outcomes differed according to baseline disease severity and did not demonstrate a direct one-to-one relationship with HPV clearance. Conclusions: Adjuvant vaccination with the nonavalent HPV vaccine is independently associated with increased HPV clearance in HPV-positive women at 1-year follow-up, irrespective of excisional treatment status. HPV clearance and histological regression represent related but distinct biological processes and should be evaluated as independent outcomes. These findings support a broader role for HPV vaccination beyond primary prevention and suggest potential clinical benefit of vaccination as an adjunctive strategy in the management of HPV-positive women.

## 1. Introduction

Persistent infection with high-risk human papillomavirus (HPV) is the central etiological factor in the development of cervical intraepithelial neoplasia (CIN) and cervical cancer [1]. Although most HPV infections are transient and cleared spontaneously, a substantial proportion persist and may progress to high-grade lesions or invasive malignancy [2,3]. The clinical challenge therefore lies not in detecting HPV infection per se, but in identifying factors that influence viral persistence and clearance [4,5].

Excisional treatment, most commonly the loop electrosurgical excision procedure (LEEP), remains the standard of care for high-grade squamous intraepithelial lesions (HSIL) [6]. While LEEP is effective in removing dysplastic tissue, it does not directly address the underlying viral infection, and HPV persistence after treatment is well documented [7,8]. Women treated for HSIL remain at increased long-term risk of recurrent disease and cervical cancer compared with the general population, highlighting the need for strategies that target viral persistence in addition to histological disease [9,10].

Prophylactic HPV vaccines have demonstrated robust efficacy in preventing HPV infection and HPV-related disease when administered prior to exposure [11]. However, increasing attention has been directed toward the potential role of HPV vaccination in HPV-positive women and in the post-treatment setting [12,13]. Accumulating evidence suggests that vaccination administered after diagnosis or excisional treatment may reduce the risk of recurrent CIN, prompting reconsideration of the traditional view that HPV vaccines are exclusively preventive [14,15,16].

Despite this growing interest, several important gaps remain. First, most studies evaluating adjuvant HPV vaccination have focused on histological recurrence as the primary outcome, whereas data on HPV viral clearance itself are limited [16,17,18]. Second, comparisons between vaccinated and unvaccinated patients are frequently confounded by disease severity, as women undergoing LEEP represent a clinically distinct subgroup with higher viral burden and more persistent infection [19,20,21]. Finally, HPV clearance and histological regression are often implicitly treated as interchangeable outcomes, despite representing distinct biological processes [22].

Recent studies, including prospective cohort analyses, have emphasized the importance of separating virological and histological endpoints and of stratifying analyses according to treatment status [23,24]. Evaluating HPV clearance as an independent outcome may provide a more direct measure of the potential immunological benefit of vaccination, particularly in real-world clinical settings [25,26].

The present study was designed to investigate the association between adjuvant vaccination with the nonavalent HPV vaccine (Gardasil 9) and HPV clearance in HPV-positive women, with and without excisional treatment. By analyzing HPV clearance and histological outcomes as independent endpoints in a large retrospective cohort with standardized follow-up, this study aims to clarify the role of HPV vaccination beyond primary prevention and to inform clinical decision-making in the management of HPV-positive women.

## 2. Materials and Methods

### 2.1. Study Design and Population

This study was conducted as a retrospective cohort analysis to evaluate the association between adjuvant HPV vaccination, excisional treatment, and post-treatment outcomes in HPV-positive women. The study was performed at a tertiary public gynecology clinic using routinely collected clinical data. No additional interventions were performed for the purposes of this study, and all diagnostic, therapeutic, and follow-up procedures were carried out as part of standard clinical practice.

### 2.2. Study Population

Women who presented to the gynecology outpatient clinic between January 2023 and December 2025 and were found to be HPV DNA-positive were screened for eligibility. Patients were included if they were aged 18 years or older and had at least 12 months of follow-up data with documented HPV testing. A total of 900 patients met the inclusion criteria and constituted the final study population. Inclusion criteria were female patients aged ≥18 years, documented HPV DNA positivity at baseline, availability of at least 12 months of follow-up data, confirmed HPV vaccination status (vaccinated or unvaccinated with Gardasil 9), documented clinical, cytological, and/or histological evaluation.

Exclusion criteria were prior HPV vaccination before study entry, incomplete HPV vaccination schedule, immunosuppressive therapy, pregnancy or postpartum status, follow-up duration < 12 months, history of cervical surgery for non-HPV-related indications, clinical assessment and diagnostic procedures. At baseline, all patients underwent standard gynecological examination. Cervical samples were collected for HPV DNA testing and liquid-based cytology. HPV testing was performed using validated molecular assays capable of detecting and genotyping high-risk HPV types. Patients with abnormal cytology or high-risk HPV positivity were referred for colposcopic evaluation. Colposcopy was performed according to internationally accepted guidelines, and targeted cervical biopsies were obtained from suspicious areas. Endocervical curettage was performed when clinically indicated.

### 2.3. Indication for LEEP and Histopathological Evaluation

Patients diagnosed with HSIL (CIN2 or higher) on biopsy underwent LEEP in accordance with current clinical guidelines. The decision to perform LEEP was based solely on cytological, colposcopic, and histopathological findings and was independent of vaccination status. Excised specimens were submitted for histopathological evaluation and classified as low-grade lesions, high-grade lesions, or malignancy.

### 2.4. HPV Vaccination Strategy

All HPV-positive patients were counseled regarding HPV vaccination irrespective of LEEP status. Vaccination was performed using the 9-valent HPV vaccine (Gardasil 9) administered in a 0–2–6 month schedule. Patients who completed all three doses were classified as vaccinated. Patients who declined vaccination for personal, socioeconomic, or access-related reasons were classified as unvaccinated. Vaccination status was verified using electronic medical records and prescription data. Importantly, all HPV vaccinations in this cohort were administered after documented HPV DNA positivity and/or abnormal cytology, as women with prior HPV vaccination before study entry were explicitly excluded.

### 2.5. Study Groups

Patients were stratified according to the following: (A) LEEP status (performed/not performed), (B) Vaccination status (vaccinated/unvaccinated). This resulted in four study groups: (1) Vaccinated with LEEP, (2) Unvaccinated with LEEP, (3) Vaccinated without LEEP, (4) Unvaccinated without LEEP.

### 2.6. Follow-Up and Outcome Definitions

Patients were followed according to standardized institutional protocols. Follow-up visits occurred at approximately 6 and 12 months. For the purposes of analysis, outcomes were assessed using HPV test results obtained closest to the 12-month follow-up point. Primary outcomes were HPV clearance, defined as the absence of detectable HPV DNA at 12 months. Secondary outcomes were HPV persistence and histological regression, persistence, or progression. HPV clearance and histological outcomes were evaluated as independent endpoints, and no direct causal relationship between the two was assumed.

### 2.7. Statistical Analysis

All statistical analysis were performed using IBM SPSS version 25 software. Continuous variables were summarized as mean ± standard deviation or median with range, as appropriate. Categorical variables were expressed as frequencies and percentages. Group comparisons were performed using Student’s *t*-test or Mann–Whitney U test for continuous variables and chi-square or Fisher’s exact test for categorical variables. Multivariable logistic regression analysis was conducted to identify independent predictors of HPV clearance. Variables entered into the model included age, vaccination status, LEEP status, baseline histological grade, and high-risk HPV type. Results were reported as odds ratios (ORs) with 95% confidence intervals (CIs). A *p*-value < 0.05 was considered statistically significant.

## 3. Results

### 3.1. Baseline Demographic and Clinical Characteristics of the Study

A total of 935 HPV-positive women were included in the final analysis. Patients were categorized according to HPV vaccination status and LEEP treatment. Of the study population, 712 patients (76.1%) were unvaccinated, while 223 patients (23.9%) completed the full three-dose Gardasil 9 vaccination schedule. Baseline demographic and clinical characteristics stratified by vaccination status are presented in Table 1. Vaccinated patients were significantly younger than unvaccinated patients (38.8 ± 7.8 vs. 42.1 ± 9.3 years, *p* < 0.001) and had a higher median parity (*p* < 0.001). Current smoking was more prevalent among vaccinated patients compared with unvaccinated patients (36.3% vs. 23.2%, *p* < 0.001). Markers of disease severity at baseline differed significantly between groups. Abnormal cytology at admission and HSIL (CIN2+) were more frequently observed in vaccinated patients (75.8% and 26.5%, respectively) compared with unvaccinated patients (42.4% and 11.8%, respectively; both *p* < 0.001). Consistent with this finding, LEEP was performed more often in the vaccinated group than in the unvaccinated group (25.1% vs. 11.5%, *p* < 0.001), reflecting appropriate clinical selection for excisional treatment.

### 3.2. Effect of Adjuvant HPV Vaccination on Viral Clearance at 12 Months

Overall HPV outcomes at 12 months according to vaccination status are summarized in Table 2. HPV clearance was observed in 66.8% of vaccinated patients, compared with 40.2% of unvaccinated patients (*p* < 0.001). Conversely, HPV persistence was significantly more frequent among unvaccinated patients (59.8%) than vaccinated patients (33.2%). These findings indicate a strong association between completion of the three-dose HPV vaccination schedule and higher HPV clearance rates at 1-year follow-up in the overall cohort.

### 3.3. Vaccination-Associated HPV Clearance Is Preserved in Both LEEP-Treated and Conservatively Managed Patients

To account for differences in baseline disease severity and treatment indication, HPV outcomes were further analyzed after stratification by LEEP status (Table 3). Among patients who underwent LEEP, HPV clearance was achieved in 73.2% of vaccinated patients, compared with 39.0% of unvaccinated patients (*p* < 0.001). Similarly, in patients managed without excisional treatment, vaccinated individuals demonstrated significantly higher HPV clearance rates than unvaccinated individuals (64.7% vs. 40.3%, *p* < 0.001). Notably, the beneficial association between vaccination and HPV clearance was consistently observed in both LEEP and non-LEEP subgroups, suggesting that the effect of vaccination on viral clearance was maintained irrespective of excisional treatment status.

### 3.4. Histological Regression Does Not Parallel Viral Clearance and Remains Dependent on Baseline Disease Severity

As shown in Table 4, histological regression was observed in 32.1% of patients in Group 1 (vaccinated + LEEP) and 25.6% in Group 2 (unvaccinated + LEEP). In contrast, higher regression rates were noted in conservatively managed patients, particularly in Group 3 (vaccinated without LEEP) compared with Group 4 (unvaccinated without LEEP). Overall, histological regression was less frequent among patients who underwent LEEP than among those managed conservatively, consistent with higher baseline lesion severity in the LEEP-treated group. Among patients who underwent LEEP, histological regression was observed in 32.1% of vaccinated patients and 25.6% of unvaccinated patients. In contrast, among patients who did not undergo LEEP, regression rates were higher, particularly in vaccinated patients (56.3%) compared with unvaccinated patients (43.0%). Across all subgroups, histological persistence and progression were more common in patients with higher baseline disease severity. Importantly, histological outcomes did not directly mirror HPV clearance rates, supporting the interpretation that viral clearance and histological regression represent related but biologically distinct processes.

### 3.5. Multivariable Analysis of Factors Associated with HPV Persistence

Multivariable logistic regression analysis was performed to identify independent factors associated with HPV persistence at 12 months (Table 5). After adjustment for age, smoking status, baseline histological severity, HPV genotype, and LEEP status, HPV vaccination emerged as an independent protective factor against HPV persistence (OR 0.34, 95% CI 0.25–0.46; *p* < 0.001). Baseline HSIL (CIN2+) (OR 1.89, 95% CI 1.31–2.72; *p* < 0.001) and HPV 16/18 positivity (OR 1.67, 95% CI 1.21–2.30; *p* = 0.002) were independently associated with increased odds of HPV persistence. LEEP status itself was not independently associated with HPV persistence after adjustment (*p* = 0.47).

## 4. Discussion

The role of prophylactic human papillomavirus (HPV) vaccination has been firmly established in primary prevention, particularly when administered prior to sexual debut. However, its potential benefit in HPV-positive women and in the post-treatment setting remains an area of active investigation and ongoing debate. The present study contributes to this evolving field by demonstrating that completion of the three-dose nonavalent HPV vaccination schedule is associated with significantly higher HPV clearance rates at 12 months, independent of baseline disease severity and excisional treatment status.

Several prior studies have suggested that HPV vaccination may exert beneficial effects beyond primary prevention [27,28]. In a prospective cohort of HPV-positive women, Pruski et al. [29]. reported a significantly lower rate of persistent HPV infection in vaccinated patients compared with unvaccinated controls, proposing vaccination as a potential element of secondary prevention. Although their cohort was relatively small, the biological signal was consistent and suggested that vaccination may enhance viral clearance rather than merely preventing new infections. Our findings extend these observations to a substantially larger cohort and reinforce the concept that vaccination may influence viral dynamics even in women with established HPV infection.

One of the central methodological challenges in this research area is the confounding effect of disease severity and treatment indication [30,31]. Patients undergoing loop electrosurgical excision procedure (LEEP) represent a clinically distinct subgroup characterized by higher-grade lesions, greater viral burden, and an intrinsically higher risk of persistence or recurrence [32,33]. Several studies examining post-conization outcomes have highlighted recurrence rates of CIN2+ ranging from 9% to 20%, driven by residual disease, persistent infection, or viral reactivation. In this context, crude comparisons between vaccinated and unvaccinated populations without adequate adjustment may be misleading [16,34,35].

In our study, vaccinated patients were more likely to present with HSIL and to undergo LEEP, reflecting appropriate clinical decision-making rather than selection bias related to vaccination. Importantly, after stratification by LEEP status and multivariable adjustment for baseline histological severity (Groups 1 and 2: excisional treatment; Groups 3 and 4: conservatively managed patients) and HPV genotype, vaccination remained independently associated with HPV clearance, whereas LEEP itself did not emerge as an independent determinant. This observation aligns with the growing consensus that excisional treatment primarily removes infected tissue but does not directly modulate systemic immune control of HPV.

The dissociation between HPV clearance and histological regression observed in our cohort merits particular attention [35]. Histological outcomes were less favorable in LEEP-treated patients regardless of vaccination status, a finding that is biologically plausible given the higher baseline lesion severity in this group. Previous studies have emphasized that viral clearance and epithelial remodeling are related yet distinct biological processes, with the former reflecting immune-mediated control and the latter dependent on tissue architecture, stromal response, and local microenvironment [36,37,38]. Our data support this distinction and caution against equating HPV negativity with immediate histological regression.

The potential mechanisms underlying the observed association between vaccination and HPV clearance remain incompletely understood [39]. Prophylactic HPV vaccines are designed to elicit neutralizing antibodies against the L1 capsid protein, thereby preventing viral entry into host cells [40]. However, emerging evidence suggests that vaccination may also enhance immune surveillance and facilitate clearance of existing infection, possibly by boosting humoral immunity and promoting antibody-mediated viral neutralization in superficial epithelial layers [41,42]. Studies demonstrating sustained antibody titers for more than a decade after vaccination lend biological plausibility to this hypothesis [43,44].

Clinical evidence supporting adjuvant vaccination in the post-treatment setting continues to accumulate. Meta-analyses have consistently shown reduced recurrence rates of CIN2+ in vaccinated women following surgical excision, with relative risk reductions of approximately 60–65% [45,46]. While many of these studies primarily focused on histological recurrence rather than HPV clearance, the convergence of evidence suggests a broader immunomodulatory effect of vaccination [47]. Our findings complement this literature by demonstrating that vaccination is associated with reduced viral persistence even when histological outcomes remain influenced by baseline disease severity.

Notably, data specifically addressing the nonavalent vaccine in HPV-positive women remain limited. Most earlier studies evaluated bivalent or quadrivalent vaccines, with heterogeneous inclusion criteria and outcome definitions. Given the broader genotype coverage of the nonavalent vaccine, its potential impact on viral clearance and reinfection dynamics may be particularly relevant in contemporary clinical practice [48,49]. Our results therefore fill an important gap by providing real-world evidence supporting the use of the nonavalent vaccine in a high-risk, HPV-positive population.

From a clinical standpoint, these findings support a paradigm shift in how HPV vaccination is conceptualized. Rather than viewing vaccination solely as a preventive intervention for HPV-naïve individuals, it may be increasingly appropriate to consider vaccination as an adjunctive strategy in the management of HPV-positive women, including those undergoing excisional treatment. This perspective is consistent with emerging recommendations that emphasize individualized risk assessment and long-term surveillance rather than a binary preventive–therapeutic distinction. Longer-term follow-up is needed to determine whether improved HPV clearance translates into sustained reductions in recurrence and cervical cancer risk.

Several limitations should be considered. The retrospective design precludes definitive causal inference, and outcome assessment relied on available follow-up data, necessitating standardized imputation strategies. Additionally, histological follow-up was not uniformly available in conservatively managed patients, reflecting real-world clinical practice rather than protocol-driven surveillance. The timing of HPV vaccination relative to excisional treatment (pre- versus post-LEEP) could not be uniformly standardized due to the retrospective nature of the study and real-world clinical practice. Therefore, vaccination timing was not analyzed as a separate variable. Additionally, causal relationships cannot be definitively established, and residual confounding cannot be completely excluded despite multivariable adjustment due to the retrospective observational design. Incorporation of immunological or virological biomarkers may help clarify why virological clearance does not always translate into immediate histological regression. Nonetheless, the large sample size, stratified analyses, and robust multivariable modeling strengthen the validity of the observed associations. As a single-center study, local clinical practices, patient characteristics, and healthcare access patterns may influence treatment decisions and vaccination uptake, potentially limiting generalizability to other healthcare settings.

## 5. Conclusions

In this large retrospective cohort of HPV-positive women, completion of the three-dose nonavalent HPV vaccination schedule was independently associated with higher HPV clearance rates at 12-month follow-up, regardless of excisional treatment status. The association between vaccination and viral clearance persisted after adjustment for baseline disease severity, indicating that the observed benefit was not merely a surrogate of treatment indication or histological stage. Importantly, HPV clearance and histological outcomes did not demonstrate a direct one-to-one relationship, underscoring that viral persistence and epithelial regression represent related but biologically distinct processes. These findings support a broader conceptualization of HPV vaccination beyond primary prevention and suggest that vaccination may serve as a clinically meaningful adjunctive strategy in the management of HPV-positive women, including those undergoing excisional treatment. Future prospective studies are warranted to further define the optimal timing and long-term impact of adjuvant HPV vaccination in secondary prevention settings. Our findings support the hypothesis that HPV vaccination may enhance immune-mediated viral control even in women with established HPV infection. These results provide a rationale for considering adjuvant HPV vaccination as a complementary component of care for HPV-positive women, particularly in specialized clinical settings.

## Figures and Tables

**Table 1:** Baseline demographic and clinical characteristics according to vaccination status.

Variable	Unvaccinated (0 Dose, *n* = 712)	Vaccinated (3 Doses, *n* = 223)	*p*-Value
Age, years (mean ± SD)	42.1 ± 9.3	38.8 ± 7.8	<0.001
Parity, median (IQR)	2 (1–4)	3 (2–4)	<0.001
Current smoker, *n* (%)	165 (23.2%)	81 (36.3%)	<0.001
Abnormal cytology at admission, *n* (%)	302 (42.4%)	169 (75.8%)	<0.001
HSIL (CIN2+) at baseline, *n* (%)	84 (11.8%)	59 (26.5%)	<0.001
LEEP performed, *n* (%)	82 (11.5%)	56 (25.1%)	<0.001

**Table 2:** HPV status at 12 months according to vaccination status.

Outcome	Unvaccinated (*n* = 712)	Vaccinated (*n* = 223)	*p*-Value
HPV clearance, *n* (%)	286 (40.2%)	149 (66.8%)	<0.001
HPV persistence, *n* (%)	426 (59.8%)	74 (33.2%)	

**Table 3:** HPV clearance at 12 months stratified by LEEP status.

Group	Unvaccinated	Vaccinated	*p*-Value
LEEP performed	*n* = 82	*n* = 56	
HPV clearance, *n* (%)	32 (39.0%)	41 (73.2%)	<0.001
HPV persistence, *n* (%)	50 (61.0%)	15 (26.8%)	
No LEEP	*n* = 630	*n* = 167	
HPV clearance, *n* (%)	254 (40.3%)	108 (64.7%)	<0.001
HPV persistence, *n* (%)	376 (59.7%)	59 (35.3%)	

**Table 4:** Histological outcomes according to LEEP and vaccination status.

Outcome	Group 1: Vaccinated + LEEP (*n* = 56)	Group 2: Unvaccinated + LEEP (*n* = 82)	Group 3: Vaccinated—No LEEP (*n* = 167)	Group 4: Unvaccinated—No LEEP (*n* = 630)
Regression, *n* (%)	18 (32.1%)	21 (25.6%)	94 (56.3%)	271 (43.0%)
Persistence, *n* (%)	27 (48.2%)	45 (54.9%)	56 (33.5%)	251 (39.8%)
Progression, *n* (%)	11 (19.7%)	16 (19.5%)	17 (10.2%)	108 (17.2%)

**Table 5:** Multivariable logistic regression analysis for HPV persistence at 12 months (*n* = 935).

Variable	OR	95% CI	*p*-Value
HPV vaccination (yes vs. no)	0.34	0.25–0.46	<0.001
LEEP performed (yes vs. no)	1.12	0.82–1.54	0.47
HSIL at baseline	1.89	1.31–2.72	<0.001
HPV 16/18 positivity	1.67	1.21–2.30	0.002
Age (per year)	0.99	0.98–1.01	0.38
Smoking	1.21	0.95–1.54	0.12

## Data Availability

The data that support the findings of this study are available from the corresponding author upon reasonable request. Due to ethical and privacy considerations, the data are not publicly available.
